# Supplementary material for: Dynamic marine viral infections and major contribution to photosynthetic processes shown by spatiotemporal picoplankton metatranscriptomes
Source: Nat Commun. 2019 Mar 12;10:1169. doi: 10.1038/s41467-019-09106-z (PMC6414667; doi:10.1038/s41467-019-09106-z)
Supplement: Supplementary file 1 — Supplementary Information [file 41467_2019_9106_MOESM1_ESM.pdf]

Dynamic marine viral infections and major contribution to photosynthetic processes shown by spatiotemporal picoplankton metatranscriptomes

Sieradzki et al.

Methods

Viral and cellular counts
Virus-like particles and bacteria/archaea were counted in whole seawater using SYBR-green dye and fluorescent microscopy in duplicates [1,2].

Secondary (heterotrophic) production
Secondary production was measured in whole seawater using ^{3}H-Thymidine and ^{3}H-Leucine[3,4]

Read processing and assembly
Raw metagenomic and metatranscriptomic reads were quality trimmed and filtered with Trimmomatic [5] version 0.33 with parameters LEADING:20 TRAILING:20 SLIDINGWINDOW:15:25. Metatranscriptomic reads were merged with PEAR [6] using the default settings, and residual ribosomal reads as well as the internal standard were removed informatically. Merged reads from each sample separately were assembled with Megahit [7] using default parameters (kmer sizes: 21, 41, 61, 81, 99). Resulting contigs smaller than 2kbp from all samples were co-assembled with Newbler version 2.9[8] (Roche) (minimum overlap 40bp minimum id 99%) and contigs larger than 2kbp from all samples were co-assembled with minimus2 [9] (minimum overlap 40bp minimum id 99%). Only 1455 contigs larger than 5 kbp were further analyzed.
Further assembly by subsampling [10] was performed on metatranscriptomes from SPOT October and POLA April separately. Each sample was subsampled at 1%, 1.5%, 2%, 5%, 10% and 20% of the original reads with different seed numbers to minimize overlap between the subsamples. Each subsample was assembled separately using metaSPAades version 3.10.1 [11] with kmer lengths 21, 33, 55, 77, 99 and 127. Reads that mapped to contigs with coverage higher than 10X were discarded from the reads dataset. The remaining reads were subsampled at 10% of the original number of reads and assembled with metaSPAdes. The process was repeated iteratively, every time discarding additional reads from the dataset, with subsamples from the reduced reads dataset of 20%, 33% and 50% of the original number of reads. Eventually all contigs longer than 1000 bp from all subsamples were concatenated into one file, dereplicated at 99% identity using cd-hit-est version 4.6 [12] and assembled with minimus2 (minimum overlap 200bp minimum id 99%). Contigs longer than 5000 bp were filtered by viral signatures as described below and searched against the existing 66 viral contigs using blastn. Only 3 additional viral contigs were identified from this dataset for a total of 69 viral contigs.

Identification and annotation of viral contigs
Viral contigs were identified by running VirSorter version 1.0.3 [13] using RefSeq on the CyVerse platform on all contigs >5 kbp and only contigs classified as category 1 or category 2 were considered. VirSorter is a well-established and reliable tool, but it has an inherent database bias and performs better on longer contigs because they are more likely to contain hallmark viral genes. Thus, all assembled contigs > 5 kbp (regardless of VirSorter results) were also ranked using VirFinder [14], which relies on k-mer signatures, and only contigs ranking higher than 0.85 were considered further. ORFs were predicted within putative viral contigs using Prodigal [15], and the amino acid sequences were searched against the nr database (August 12th 2016) using blastp [16] with a maximum E-value 10^{-5}. VirFinder-identified contigs with at least one gene whose best blast hit was a viral gene were added to the VirSorter-identified contigs, and generally we identified at least 2 ORFs with a best blast hit to a viral gene (sup. table S3). This final set of contigs were visually verified to be non-chimeric by consistent read recruitment along the each

contig in samples where that contig had reads mapped to it. Since most contigs were active only ephemerally, a total of 238 recruitment plots were inspected including recruitment to cyanophage and pelagiphage reference genomes.
Quality filtered metagenomic and metatranscriptomic reads were mapped back to the viral contigs with Bowtie2 version 2.2.6 [17] using the default settings and the expression patterns were identified and visualized with Anvi'o [18] version 2.1.0.

Microbial community composition analysis
The V4-V5 regions of the 16S-rRNA coding gene were amplified from DNA and cDNA from all samples using the 515(N)-F and 926-R primers, and sequenced on an Illumina MiSeq 2x300 bp (UC Davis genome center) along with a negative controls and even and staggered mock communities [19].
The ends of resulting reads were trimmed with PRINSEQ [20] to a quality score higher than 20. The trimmed reads were merged with USEARCH7 [21] allowing for 3 mismatches in the overlap region. Retained assembled reads were grouped as amplicon single variants with the Minimum Entropy Decomposition (MED-typing) pipeline [22]. Bray-Curtis dissimilarity was calculated with R package vegan [23].

Analysis of PS-II D1 protein sequences
A curated set of PS-II D1 amino acid sequences of myoviruses, podoviruses, cyanobacteria and eukaryotes (chloroplast) from Pfam[24] and RefSeq release 80 was downloaded. All sequences of marine viral PS-II D1 were retained in addition to sequences of bacterial and eukaryotic taxa that were identified in the 16S-rRNA community composition. One of the assembled contigs contained a *psbA* gene coding for PS-II D1. The translated amino acid sequences were added to the set of proteins.
Merged reads from the metatranscriptomes and unmerged forward reads from the metagenomes were aligned with blastx [16] against this set demanding an e-value of 10^{-5}. The reads that passed this filter were translated using bioPython [25] into amino acids according to the reading frame indicated by the blastx start and end values.
Following the protocol used in Ignacio-Espinoza et al. [26], a total of 158 sequences were aligned with MAFFT version 7.305b [27] with parameters set to globalpair, gap open penalty 1.5, gap extension penalty 0.5 and scoring matrix BLOSUM30. Informative blocks were identified using Gblocks [28] version 0.91b with a minimum block length 5, blocks represent at least half of the sequences and allowing gaps (b3=50, b4=5, b5=h). The blocks were used to build a maximum likelihood phylogenetic tree using RAxML [29] (best of 20 trees, gamma model and WAG substitution matrix) visualized by the IToL web interface [30]. A hidden Markov Model (HMM) of the same set was also built with hmmer 3.0 [31]. The translated metagenomics and metatranscriptomics amino acid sequences were searched using the HMM and a threshold of e-value 10^{-5}. A total of 190,928 translated metatranscriptomics reads and 72,292 metagenomics reads from all samples remained after this step. Those reads were locally aligned to the HMM using hmmer 3.0 function hmmalign and placed into the phylogenetic tree using pplacer version v1.1.alpha17 [32] (sup. figs. S5, S7).

Supplementary Table 1: Accession numbers of Gp23 proteins used to build the Gp23 HMM and the maximum likelihood tree used in pplacer

Phage	Gp23 accession number
Synechococcus phage S-ShM2	YP_004322790
Cyanophage P-TIM40	YP_009188211
Synechococcus phage ACG-2014h	YP_009008247
Synechococcus phage S-PM2	YP_195142
Prochlorococcus phage Syn1	YP_004324495
Sinorhizobium phage phiM12	YP_009142975
Deftia phage phiW-14	YP_003358893
Erwinia phage phiEa2809	YP_009147619
Salmonella phage Det7	YP_009140190
Dickeya virus Limestone	YP_007237460
Shigella phage Ag3	YP_003358645
Vibrio phage KVP40	NP_899609.1
Enterobacteria phage T4	NP_049787.1
Escherichia phage 121Q	YP_009102190
Sinorhizobium phage phiM9	YP_009189482
Campylobacter virus CP21	YP_007005149
Synechococcus phage S-SSM7	YP_004324201
Synechococcus phage S-SM2	YP_004322275
Prochlorococcus phage P-SSM2	YP_214367
Synechococcus phage S-CRM01	YP_004508477
Prochlorococcus phage P-HM1	YP_004322545
Prochlorococcus phage P-HM2	YP_004323491
Synechococcus phage Syn19	YP_004323954
Synechococcus phage syn9	YP_717802
Prochlorococcus phage P-SSM4	YP_214669
Prochlorococcus phage Syn33	YP_004323731
Prochlorococcus phage P-SSM7	YP_004324955
Synechococcus phage S-SM1	YP_004323024
Prochlorococcus phage P-RSM4	YP_004323268
Synechococcus phage S-SSM5	YP_004324729

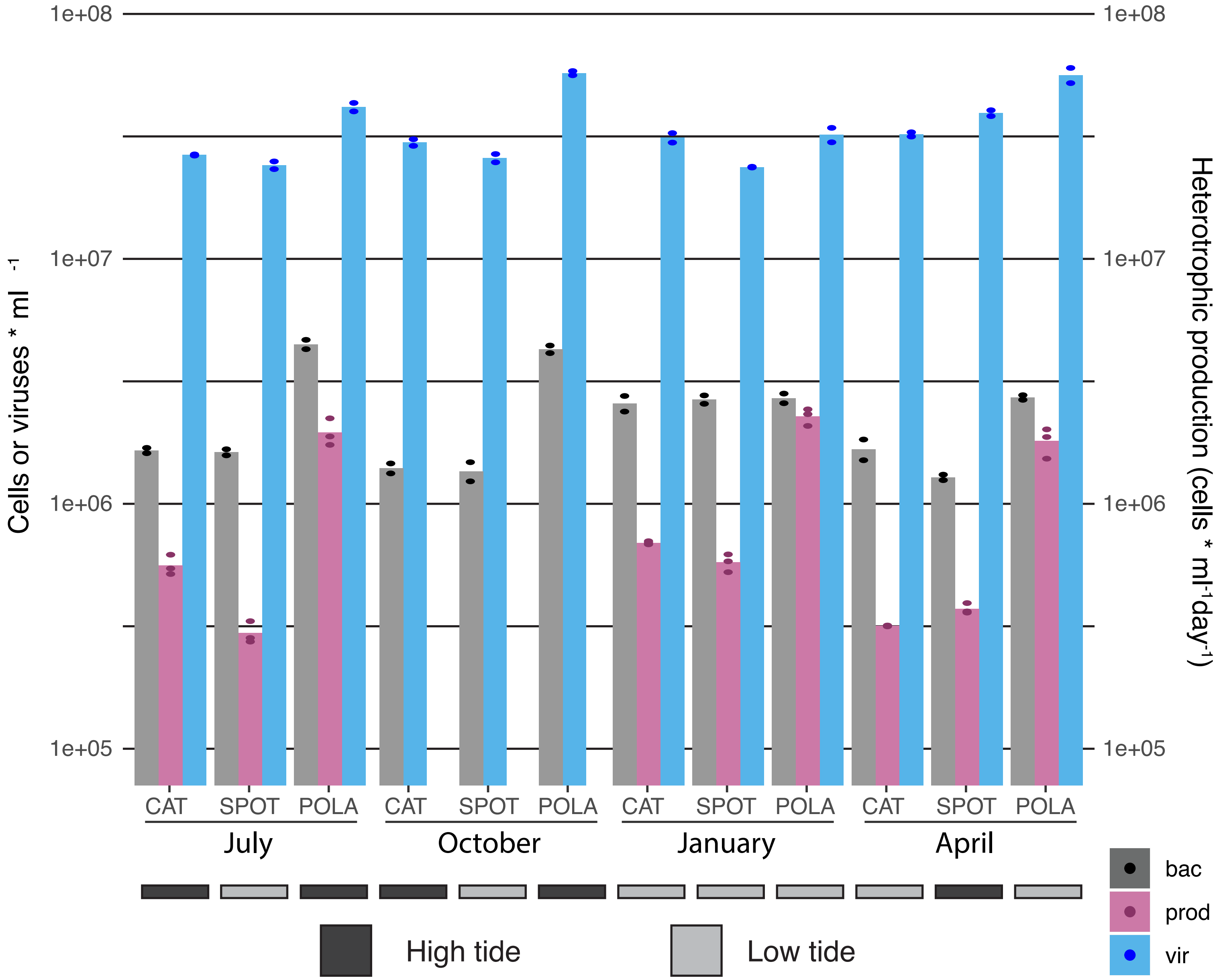

Supplementary Figure 1: Biotic ecological parameters per site and season
Virus-like particles or bacterial+archaeal cells per ml and secondary production (cells per ml per day) coupled to tide direction by site and date. There is no production data for October 2012. Raw data is plotted on top of the bars.

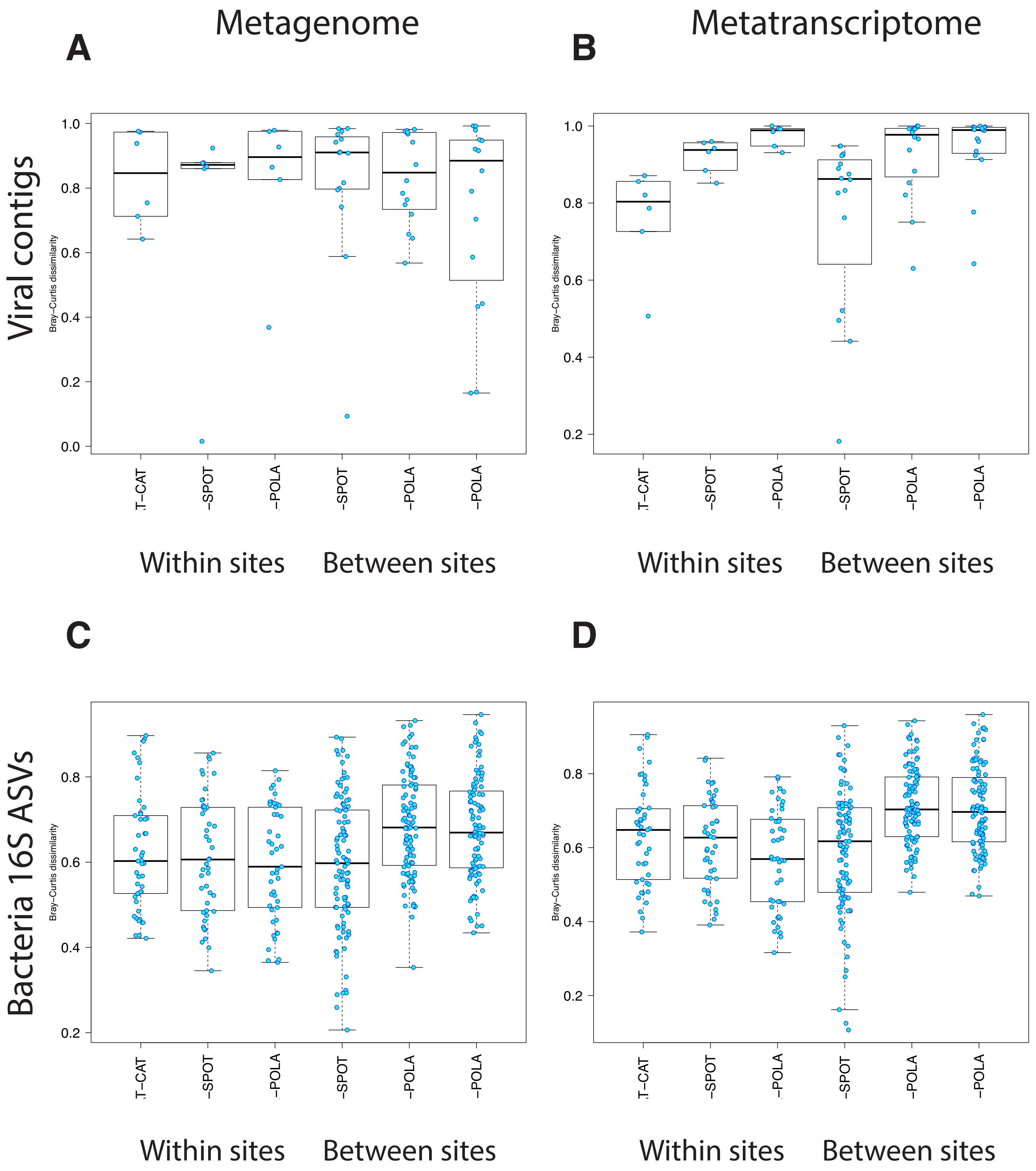

Supplementary Figure 2: Active-viral and bacterial community composition variation
Distribution of Bray-Curtis dissimilarity based on mean coverage of the active viral contigs (N=16 between sites, N=6 within sites) **(A)** using metagenomic reads recruitment, **(B)** using metatranscriptomic reads recruitment, and of the bacterial and archaeal community based on 16S-rRNA ASVs (amplicon single variants) relative abundance (N=100 between sites, N=45 within sites) **(C)** using amplicons from DNA, and **(D)** using amplicons from cDNA. The box represents the 25-75% of the samples, the line inside it denotes the median and the whiskers represent a 95% confidence interval. Raw data is plotted on top of the box plots.

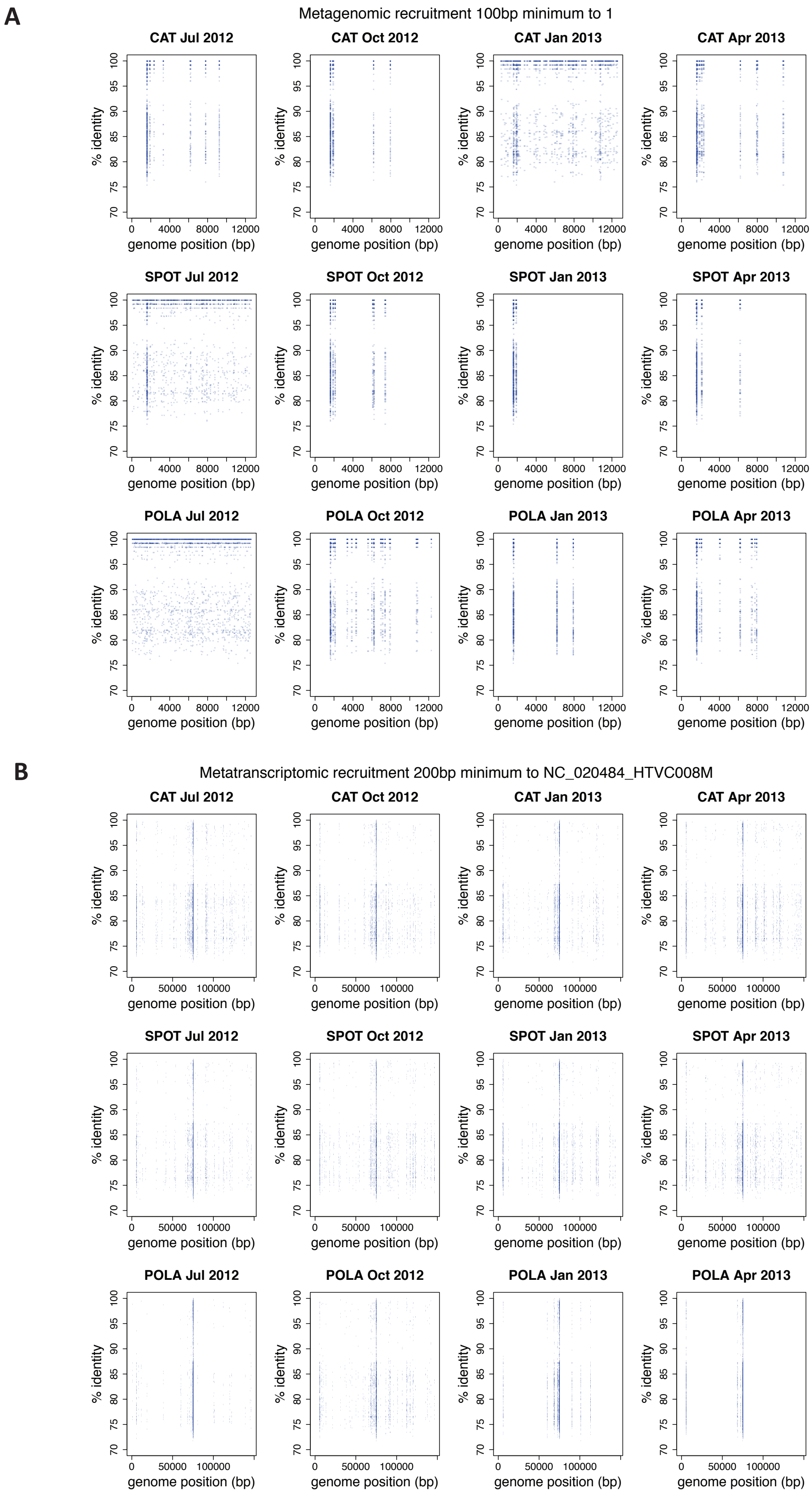

Supplementary Figure 3: Artifacts in recruitment may affect mean coverage
Examples of localized recruitment to short regions **(A)** of metagenomics reads to an assembled viral contig and **(B)** of metatranscriptomics reads to the published genome of pelagiphage HTVC008M. This artifact may cause mean coverage to appear high but in reality, indicates a very highly conserved short sequence within the contig that triggers non-specific recruitment. An example of continuous recruitment throughout the contig can be seen in **(A)** SPOT and POLA Jul 2012.

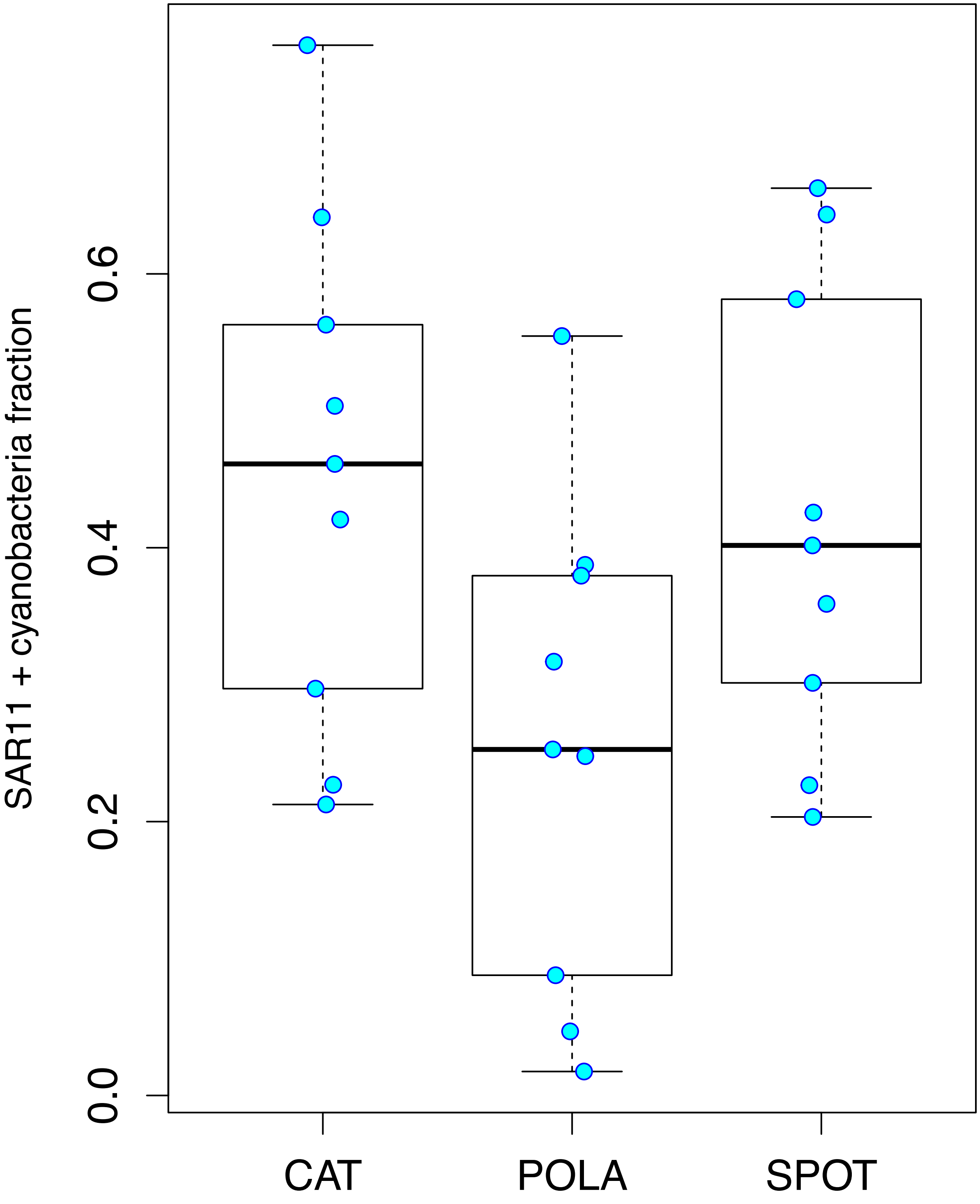

Supplementary Figure 4: SAR11 and cyanobacteria are less abundant in the Port of LA
Fraction of the prokaryotic community representing SAR11 and cyanobacteria at all three sites over 10 quarterly cruises (July 2012 to October 2014). The box represents the 25-75% of the samples, the line inside it denotes the median and the whiskers represent a 95% confidence interval. Raw data is plotted on top of the box plots.

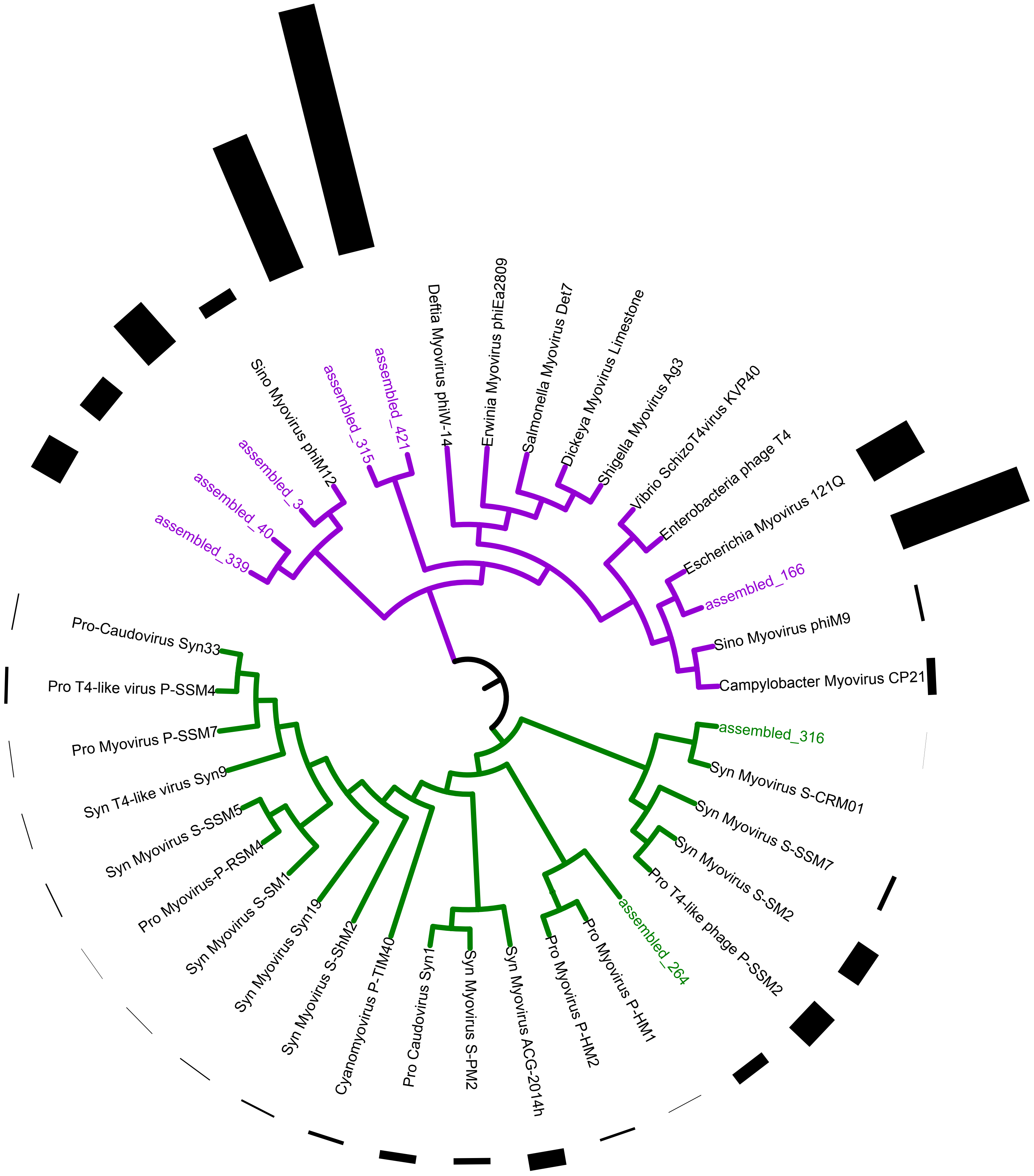

Supplementary Figure 5: Maximum likelihood dendrogram of Gp23 proteins.
Green branches and purple branches denote cyanomyovirus clades and myoviruses of heterotrophic bacteria respectively. Labels of open reading frames from assembled contigs are colored according to the clade the were placed in. Bar charts outside the dendrogram indicate cumulative mean coverage of a protein across all samples. Cyanophages have a much lower coverage than heterophages.

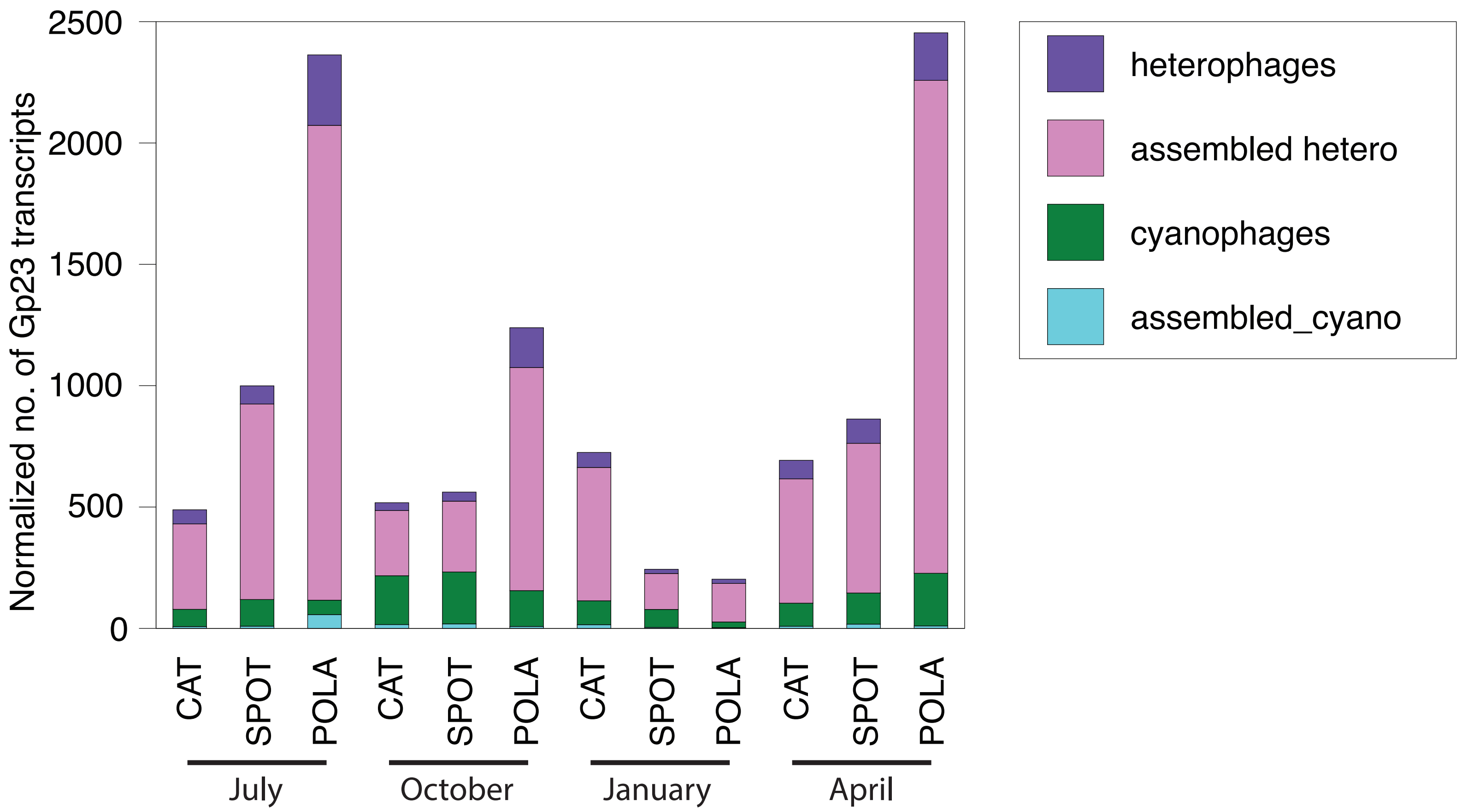

Supplementary Figure 6: Expression of capsid proteins is dominated by heterophages
Number of transcripts of Gp23, normalized to sample sequencing depth, from assembled contigs and published cyanophage genomes (accession numbers in sup. table S5) over time and space. Expression was measured as metatranscriptomic reads mapped to proteins using HMMsearch and HMMalign with an e-value cutoff of 10-5. Expression is persistent and comparable between samples for most phages.

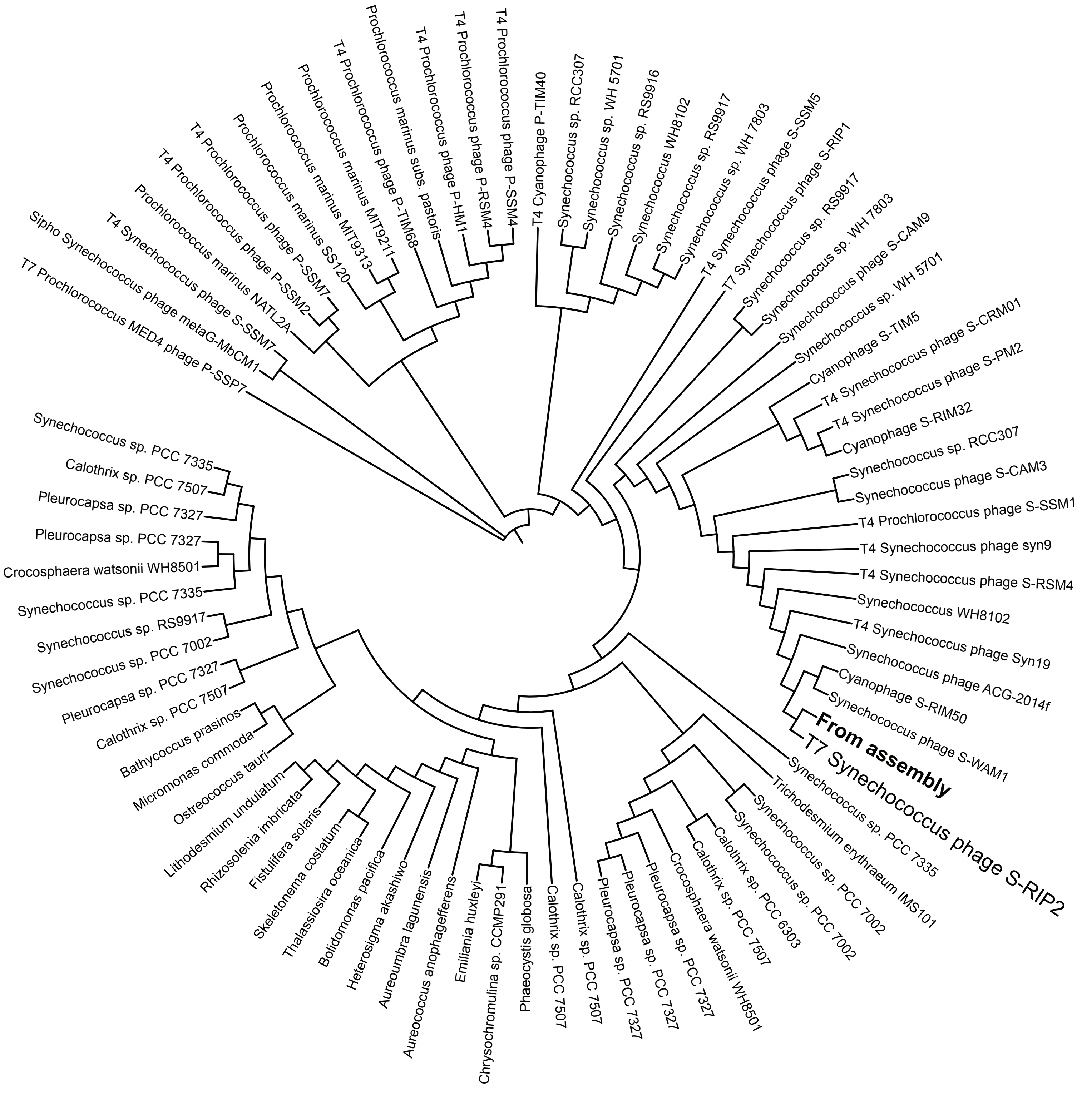

Supplementary Figure 7: Assembled cyanophage is closely related to Synechococcus podovirus

maximum-likelihood tree (branch lengths ignored) of PS-II D1 protein from assembly and marine viruses, cyanobacteria and picoeukaryotes

.

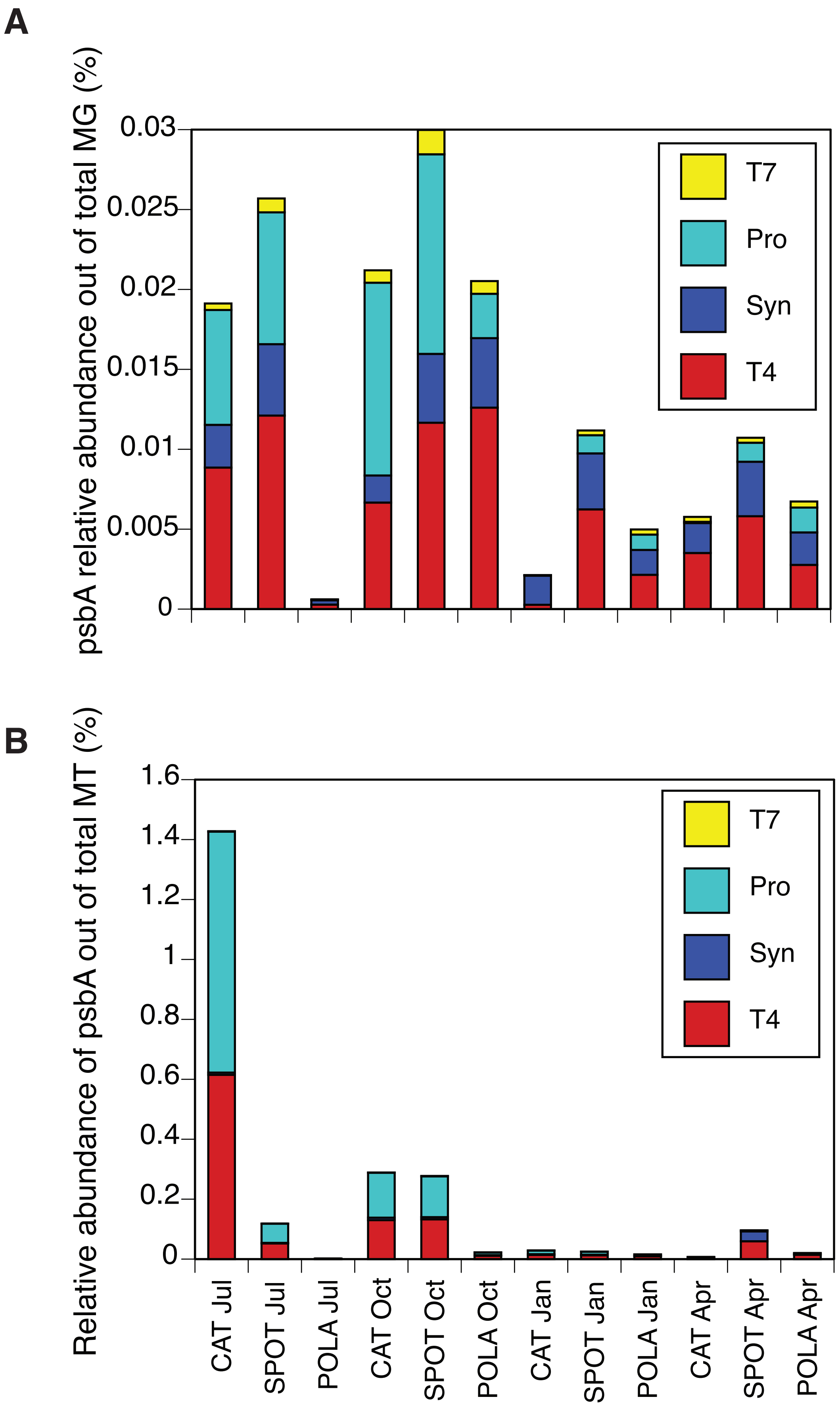

Supplementary Figure 8: Relative abundance and expression of PS-II
Distribution of psbA relative abundance by domain (T4-like cyanomyoviruses, T7-like cyanopodoviruses, Synechococcus, Prochlorococcus) out of the entire (A) metagenome and (B) metatranscriptome.

References

1. Noble, R. T. & Fuhrman, J. A. Use of SYBR Green I for rapid epifluorescence counts of marine viruses and bacteria. *Aquat. Microb. Ecol.* **14**, 113–118 (1998).
2. Patel, A. *et al.* Virus and prokaryote enumeration from planktonic aquatic environments by epifluorescence microscopy with SYBR Green I. *Nat. Protoc.* **2**, 269–276 (2007).
3. Fuhrman, J. A. & Azam, F. Bacterioplankton secondary production estimates for coastal waters of british columbia, antarctica, and california. *Appl. Environ. Microbiol.* **39**, 1085–1095 (1980).
4. Kirchman, D., K'nees, E. & Hodson, R. Leucine incorporation and its potential as a measure of protein synthesis by bacteria in natural aquatic systems. *Appl. Environ. Microbiol.* **49**, 599–607 (1985).
5. Bolger, A. M., Lohse, M. & Usadel, B. Trimmomatic: a flexible trimmer for Illumina sequence data. *Bioinformatics* **30**, 2114–2120 (2014).
6. Zhang, J., Kobert, K., Flouri, T. & Stamatakis, A. PEAR: a fast and accurate Illumina Paired-End reAd mergeR. *Bioinformatics* **30**, 614–620 (2014).
7. Li, D., Liu, C.-M., Luo, R., Sadakane, K. & Lam, T.-W. MEGAHIT: an ultra-fast single-node solution for large and complex metagenomics assembly via succinct de Bruijn graph. *Bioinformatics* **31**, 1674–1676 (2015).
8. Margulies, M. *et al.* Genome sequencing in microfabricated high-density picolitre reactors. *Nature* **437**, 376–380 (2005).
9. Sommer, D. D., Delcher, A. L., Salzberg, S. L. & Pop, M. Minimus: a fast, lightweight genome assembler. *BMC Bioinformatics* **8**, 64 (2007).
10. Hug, L. A. *et al.* Critical biogeochemical functions in the subsurface are associated with bacteria from new phyla and little studied lineages. *Environ. Microbiol.* **18**, 159–173

(2016).

11. Nurk, S., Meleshko, D., Korobeynikov, A. & Pevzner, P. A. metaSPAdes: a new versatile metagenomic assembler. *Genome Res.* **27**, 824–834 (2017).

12. Fu, L., Niu, B., Zhu, Z., Wu, S. & Li, W. CD-HIT: accelerated for clustering the next-generation sequencing data. *Bioinformatics* **28**, 3150–3152 (2012).

13. Roux, S., Enault, F., Hurwitz, B. L. & Sullivan, M. B. VirSorter: mining viral signal from microbial genomic data. *PeerJ* **3**, e985 (2015).

14. Ren, J., Ahlgren, N. A., Lu, Y. Y., Fuhrman, J. A. & Sun, F. VirFinder: a novel k-mer based tool for identifying viral sequences from assembled metagenomic data. *Microbiome* **5**, 69 (2017).

15. Hyatt, D. *et al.* Prodigal: prokaryotic gene recognition and translation initiation site identification. *BMC Bioinformatics* **11**, 119 (2010).

16. Camacho, C. *et al.* BLAST+: architecture and applications. *BMC Bioinformatics* **10**, 421 (2009).

17. Langmead, B. & Salzberg, S. L. Fast gapped-read alignment with Bowtie 2. *Nat. Methods* **9**, 357–359 (2012).

18. Eren, A. M. *et al.* Anvi'o: an advanced analysis and visualization platform for 'omics data. *PeerJ* **3**, e1319 (2015).

19. Parada, A. E., Needham, D. M. & Fuhrman, J. A. Every base matters: assessing small subunit rRNA primers for marine microbiomes with mock communities, time series and global field samples. *Environ. Microbiol.* **18**, 1403–1414 (2016).

20. Schmieder, R. & Edwards, R. Quality control and preprocessing of metagenomic datasets. *Bioinformatics* **27**, 863–864 (2011).

21. Edgar, R. C. Search and clustering orders of magnitude faster than BLAST. *Bioinformatics*

26, 2460–2461 (2010).

22. Eren, A. M. *et al.* Minimum entropy decomposition: unsupervised oligotyping for sensitive partitioning of high-throughput marker gene sequences. *ISME J.* **9**, 968–979 (2015).

23. Oksanen, J. *et al.* The vegan package. *Community ecology package. [http://r-forge. r-project. org/projects/vegan/]* (2008).

24. Finn, R. D. *et al.* The Pfam protein families database: towards a more sustainable future. *Nucleic Acids Res.* **44**, D279–85 (2016).

25. Cock, P. J. A. *et al.* Biopython: freely available Python tools for computational molecular biology and bioinformatics. *Bioinformatics* **25**, 1422–1423 (2009).

26. Ignacio-Espinoza, J. C. & Sullivan, M. B. Phylogenomics of T4 cyanophages: lateral gene transfer in the 'core' and origins of host genes: Molecular evolution of T4 myoviruses. *Environ. Microbiol.* **14**, 2113–2126 (2012).

27. Katoh, K. & Standley, D. M. MAFFT multiple sequence alignment software version 7: improvements in performance and usability. *Mol. Biol. Evol.* **30**, 772–780 (2013).

28. Castresana, J. Selection of conserved blocks from multiple alignments for their use in phylogenetic analysis. *Mol. Biol. Evol.* **17**, 540–552 (2000).

29. Stamatakis, A. RAxML version 8: a tool for phylogenetic analysis and post-analysis of large phylogenies. *Bioinformatics* **30**, 1312–1313 (2014).

30. Letunic, I. & Bork, P. Interactive tree of life (iTOL) v3: an online tool for the display and annotation of phylogenetic and other trees. *Nucleic Acids Res.* **44**, W242–5 (2016).

31. Johnson, L. S., Eddy, S. R. & Portugaly, E. Hidden Markov model speed heuristic and iterative HMM search procedure. *BMC Bioinformatics* **11**, 431 (2010).

32. Matsen, F. A., Kodner, R. B. & Armbrust, E. V. pplacer: linear time maximum-likelihood and Bayesian phylogenetic placement of sequences onto a fixed reference tree. *BMC*

Bioinformatics **11**, 538 (2010).
