# Supplementary material for: Dynamic marine viral infections and major contribution to photosynthetic processes shown by spatiotemporal picoplankton metatranscriptomes
Source: Nat Commun. 2019 Mar 12;10:1169. doi: 10.1038/s41467-019-09106-z (PMC6414667; doi:10.1038/s41467-019-09106-z)
Supplement: Supplementary file 3 — Description of Additional Supplementary Files [file 41467_2019_9106_MOESM3_ESM.pdf]

Description of Additional Supplementary Files

File Name: Supplementary Data 1
Description: Mean coverage and length (bp) of assembled viral contigs and reference genomes

File Name: Supplementary Data 2
Description: Annotations of open reading frames (ORFs) in the assembled viral contigs

File Name: Supplementary Data 3
Description: VirSorter and VirFinder identification/ranking of the 69 coassembled viral contigs and number of viral ORFs verified by blastp to the NR database
