# Supplementary material for: Dynamic marine viral infections and major contribution to photosynthetic processes shown by spatiotemporal picoplankton metatranscriptomes
Source: Nat Commun. 2019 Mar 12;10:1169. doi: 10.1038/s41467-019-09106-z (PMC6414667; doi:10.1038/s41467-019-09106-z)
Supplement: Supplementary file 7 — Reporting Summary [file 41467_2019_9106_MOESM7_ESM.pdf]

nature research

Corresponding author(s): Jed A. Fuhrman

Last updated by author(s): Feb 4, 2019

Reporting Summary

Nature Research wishes to improve the reproducibility of the work that we publish. This form provides structure for consistency and transparency in reporting. For further information on Nature Research policies, see Authors & Referees and the Editorial Policy Checklist.

Statistics

For all statistical analyses, confirm that the following items are present in the figure legend, table legend, main text, or Methods section.

n/a	Confirmed	
☐	☒	The exact sample size (*n*) for each experimental group/condition, given as a discrete number and unit of measurement
☐	☒	A statement on whether measurements were taken from distinct samples or whether the same sample was measured repeatedly
☒	☐	The statistical test(s) used AND whether they are one- or two-sided<br>*Only common tests should be described solely by name; describe more complex techniques in the Methods section.*
☒	☐	A description of all covariates tested
☒	☐	A description of any assumptions or corrections, such as tests of normality and adjustment for multiple comparisons
☐	☒	A full description of the statistical parameters including central tendency (e.g. means) or other basic estimates (e.g. regression coefficient) AND variation (e.g. standard deviation) or associated estimates of uncertainty (e.g. confidence intervals)
☒	☐	For null hypothesis testing, the test statistic (e.g. *F*, *t*, *r*) with confidence intervals, effect sizes, degrees of freedom and *P* value noted<br>*Give P values as exact values whenever suitable.*
☒	☐	For Bayesian analysis, information on the choice of priors and Markov chain Monte Carlo settings
☒	☐	For hierarchical and complex designs, identification of the appropriate level for tests and full reporting of outcomes
☒	☐	Estimates of effect sizes (e.g. Cohen's *d*, Pearson's *r*), indicating how they were calculated

Our web collection on statistics for biologists contains articles on many of the points above.

Software and code

Policy information about availability of computer code

Data collection	No software was used
Data analysis	Trimmomatic version 0.33, PEAR version 0.9.8, Megahit version 1.0.4 beta, Newbler version 2.9, minimus2 version 2.0.8, seqmagick version 0.5.0, seqtk version 1.0, metaSPAades version 3.10.1, cd-hit-est version 4.6, BLAST version BLAST 2.2.30, VirSorter version 1.0.3, VirFinder version 1.1, Prodigal version 2.6.2, RStudio base package version 1.1.456, Bowtie2 version 2.2.6, Anvi'o version 2.1.0, PRINSEQ version 0.20.4, Usearch version 7, mothur version 1.38.0, SILVA version 119, R package vegan version 2.5-3, biopython version 1.72, MAFFT version 7.305b, Gblocks version 0.91b, RAxML version 8.2.5, hmmer version 3.0, pplacer version v1.1.alpha17, No custom code was used

For manuscripts utilizing custom algorithms or software that are central to the research but not yet described in published literature, software must be made available to editors/reviewers. We strongly encourage code deposition in a community repository (e.g. GitHub). See the Nature Research guidelines for submitting code & software for further information.

Data

Policy information about availability of data

All manuscripts must include a data availability statement. This statement should provide the following information, where applicable:

- Accession codes, unique identifiers, or web links for publicly available datasets
- A list of figures that have associated raw data
- A description of any restrictions on data availability

All raw data can be found on EMBL-ENA under project number PRJEB12234 [https://www.ncbi.nlm.nih.gov/bioproject/PRJEB12234]. Raw metatranscriptomics sequences accession numbers are ERS1864892-ERS1864903 [https://www.ncbi.nlm.nih.gov/Traces/study/?acc=ERP013687], and negative control library sequences accession number is ERR2089009 [https://trace.ncbi.nlm.nih.gov/Traces/sra/sra.cgi?run=ERR2089009]. Raw metagenomic sequences accession numbers are ERS1869885-ERS1869896 [https://trace.ncbi.nlm.nih.gov/Traces/sra/sra.cgi?study=ERP013687] and negative control accession number is ERS1872073 [https://

www.ncbi.nlm.nih.gov/Traces/study/?acc=ERS1872073&go=go]. The 69 assembled viral contigs can be found in Genbank under Bioproject PRJNA472807 [https://www.ncbi.nlm.nih.gov/bioproject/PRJNA472807] accession numbers QKOA01000001-QKOA01000069 [https://www.ncbi.nlm.nih.gov/nuccore/QKOA00000000.1].

Field-specific reporting

Please select the one below that is the best fit for your research. If you are not sure, read the appropriate sections before making your selection.

☐ Life sciences ☐ Behavioural & social sciences ☒ Ecological, evolutionary & environmental sciences

For a reference copy of the document with all sections, see nature.com/documents/nr-reporting-summary-flat.pdf

Ecological, evolutionary & environmental sciences study design

All studies must disclose on these points even when the disclosure is negative.

Study description	This study aimed to explore seasonal differences in marine microbial communities, including viruses, over a spatial gradient of human impact. We focused on bacteria infected by phages via metatranscriptomes of the free-living cellular fraction of the community.
Research sample	Each sample consisted of 20 liters of surface seawater filtered sequentially through: 80u mesh, 1u glass prefilter, 0.2u filter. The 0.2u filter represents most of the free-living microbes including bacteria, archaea and picoeukaryotes. Particle-attached prokaryotes and larger eukaryotes were excluded by the mesh and prefilter.
Sampling strategy	Seawater was collected by bucket after washing the bucket, funner and cubitainers with sample water 3 times. Due to the high costs of metagenomics and metatranscriptomics we opted for collection of large samples without replication as opposed to small samples with replication.
Data collection	Subsampling for cell/virus counts, secondary production and nutrient analysis was performed the day of the cruise by Fuhrman lab members. Cell/virus duplicate slides were prepared the same day by David Needham and counted the next day by Erin Fichot, secondary production by leucine/thymidine incorporation triplicates were incubated the same day and measured the next day by Ella Sieradzki. Triplicate subsamples for nutrient analysis were kept in -80 degrees C until analysis.
Timing and spatial scale	Samples were collected from 3 sites across the San Pedro Channel: the Port of Los Angeles, the San Pedro Ocean Time-series and Two Harbors covering a transect of 37km. These sites coincide with different levels of human impact: high, pristine and low respectively. Collection was performed on 4 dates representing different seasons: July 15 2012, October 19 2012, January 9 2013 and April 24 2013.
Data exclusions	No data was excluded from the analyses.
Reproducibility	We did not attempt to reproduce the experiments.
Randomization	Samples were processed on the day of collection so as to produce results as true to actual ocean conditions as possible, therefore they were grouped by sampling date.
Blinding	For nutrient measurements the facility that provided the analysis did not know what the sample names correspond to. The same applies for the sequencing facility.

Did the study involve field work? ☒ Yes ☐ No

Field work, collection and transport

Field conditions	July 2012: overcast at POLA and SPOT, sunny at CAT, water temperature 16-19C, samples collected between 7:30 and 10 am.<br>October 2012: overcast at all sites, water temperature 20-20.6C, samples collected between 8:40 and 11 am.<br>January 2013: sunny at POLA, overcast at SPOT and CAT, water temperature 14-15C, samples collected between 8:20 and 10:20 am.<br>April 2013: overcast at all sites, water temperature 14.5-6C, samples collected between 9:30 and 11:30 am.
Location	Port of Los Angeles (POLA, 33.71N -118.254W), the San Pedro Ocean Time-series (SPOT, 33.55N -118.4W) and Two Harbors, Santa Catalina Island (CAT, 33.454N -118.475W)<br>Water depth 5m (mixed layer)
Access and import/export	Sampling sites were accessed by 54ft diving boat. No permits are necessary for collection of seawater.
Disturbance	No disturbance was caused by this study.

Reporting for specific materials, systems and methods

We require information from authors about some types of materials, experimental systems and methods used in many studies. Here, indicate whether each material, system or method listed is relevant to your study. If you are not sure if a list item applies to your research, read the appropriate section before selecting a response.

Materials & experimental systems

n/a	Involved in the study	
☒	☐	Antibodies
☒	☐	Eukaryotic cell lines
☒	☐	Palaeontology
☒	☐	Animals and other organisms
☒	☐	Human research participants
☒	☐	Clinical data

Methods

n/a	Involved in the study	
☒	☐	ChIP-seq
☒	☐	Flow cytometry
☒	☐	MRI-based neuroimaging
